# *Pneumocystis *cell wall β-glucan stimulates calcium-dependent signaling of IL-8 secretion by human airway epithelial cells

**DOI:** 10.1186/1465-9921-11-95

**Published:** 2010-07-13

**Authors:** Eva M Carmona, Jeffrey D Lamont, Ailing Xue, Mark Wylam, Andrew H Limper

**Affiliations:** 1 From the Thoracic Diseases Research Unit, Division of Pulmonary Critical Care and Internal Medicine, Department of Medicine Mayo Clinic and Foundation, Rochester, Minnesota, 55905, USA

## Abstract

**Background:**

Respiratory failure secondary to alveolar inflammation during *Pneumocystis *pneumonia is a major cause of death in immunocompromised patients. Neutrophil infiltration in the lung of patients with *Pneumocystis *infection predicts severity of the infection and death. Several previous studies indicate that airway epithelial cells release the neutrophil chemoattractant proteins, MIP-2 (rodents) and IL-8 (humans), in response to *Pneumocystis *and purified *Pneumocystis *cell wall β-glucans (PCBG) through the NF-κB-dependent pathway. However, little is known about the molecular mechanisms that are involved in the activation of airway epithelium cells by PCBG resulting in the secretion of IL-8.

**Method:**

To address this, we have studied the activation of different calcium-dependent mitogen-activated protein kinases (MAPKs) in 1HAEo^- ^cells, a human airway epithelial cell line.

**Results:**

Our data provide evidence that PCBG induces phosphorylation of the MAPKs, ERK, and p38, the activation of NF-κB and the subsequently secretion of IL-8 in a calcium-dependent manner. Further, we evaluated the role of glycosphingolipids as possible receptors for β-glucans in human airway epithelial cells. Preincubation of the cells with D-*threo*-1-phenyl-2-decanoylamino-3-morpholino-1-propanol (PDMP) a potent inhibitor of the glycosphingolipids synthesis, prior to PCBG stimulation, significantly decreased IL-8 production.

**Conclusion:**

These data indicate that PCBG activates calcium dependent MAPK signaling resulting in the release of IL-8 in a process that requires glycosphingolipid for optimal signaling.

## Introduction

*Pneumocystis *pneumonia is an opportunistic infection, caused by *Pneumocystis jirovecii *that predominantly affects immunosuppressed patients, including those with AIDS and malignancy. With the introduction of the highly active retroviral therapy (HAART) the incidence of *Pneumocystis *pneumonia among the HIV-infected patients has decreased significantly, but still remains among the most common severe opportunistic infection in this group of patients [1]. In addition, in non-HIV immunocompromised patients *Pneumocystis *infection is associated with substantially greater morbidity and mortality when compared with HIV-positive population despite the available medication [2].

It has been postulated that one reason for the differential mortality rates between the two groups is based on the differing abilities to mount inflammatory responses in the face of infection; with non-HIV-infected patients having a more robust inflammatory response against the organism is elicited compared to HIV-infected individuals. Indeed, this exuberant inflammatory reaction towards the organism has been shown to be more harmful to the host than the organism burden itself [3-5]. Polymorphonuclear neutrophils (PMN) are one of the major components of the lung inflammatory reaction seen in patients affected with *Pneumocystis *pneumonia, though CD8 cells and other cells are known to participate as well [6-8]. Moreover, it has been documented that the degree of neutrophil infiltration in the lung of these patients can serve as a marker of the severity of respiratory failure and death [3-5,9]. From theses observations, we have further postulated that a balanced inflammatory response is necessary to successfully control *Pneumocystis *infection.

*Pneumocystis *organisms are present within the alveolus in at least two different developmental stages, namely the trophic form and the cyst. The trophic form attaches firmly to the alveolar epithelium, in a process that stimulates organism proliferation [10]. The cyst form is characterized by a thick β-glucan rich cell wall, which recent studies have implicated as a major initiator of lung inflammation during *Pneumocystis *infection [11,12]. However, the molecular mechanisms by which β-glucans induce this exaggerated airway inflammatory response have not yet been fully elucidated.

Airway epithelial cells actively participate in the immune response during infection, not only by recognizing the microorganisms, but also by initiating appropriate signal transduction pathways that will lead to the production of a variety of cytokines and chemokines involved in the recruitment of inflammatory cells to the site of infection. In the case of *Pneumocystis*, various studies have demonstrated that *Pneumocystis *organisms closely associate with airway epithelial cells; supporting the tenant that binding of the organism to airway epithelial cells is an integral component in the establishment of infection [13,14]. While *Pneumocystis *trophic forms bind preferentially to Type I alveolar cells, *Pneumocystis *cysts and degraded components can be found in expectorated sputum [15]. Thus, *Pneumocystis *components such as glucan have ample opportunity to interact with epithelial cells in the lower respiratory tract.

Our group has demonstrated that fungal β-glucans in the wall of *Pneumocystis *induce NF-κB translocation and TNF-α production in macrophages following contact with the phagocyte [16]. In addition, we have also demonstrated that *Pneumocystis *β-glucans (PCBG) stimulate rat airway epithelial cells to secrete macrophage inflammatory protein-2 (MIP-2) through NF-κB dependent mechanisms [17,18]. However, the events through which PCBG initiate airway epithelial cells activation remain unclear. Various bacterial pathogens such as *Salmonella *and *Pseudomonas *species activate epithelial cells by increasing intracellular calcium concentrations [19,20]. For instance, during pseudomonal infection, superficial interactions of the microbe with airway epithelial cells are sufficient to induce changes in calcium influx and subsequently stimulate NF-κB-dependent gene expression [19]. We, therefore, hypothesized that following binding of PCBG to airway epithelial cells, the epithelial cells are stimulated to express pro-inflammatory responses by inducing changes in cytosolic calcium influx. These changes in intracellular calcium subsequently activate major signal transduction pathways that eventually lead to cytokine secretion by airway epithelial cells.

Fungal adhesion to host tissues is an integral step for colonization and subsequent infection [10,21,22]. Histological studies of *Pneumocystis *infected patients and animals demonstrate intimate association of *Pneumocystis *organisms with alveolar epithelial cells [13]. Many receptors have been proposed to bind *Pneumocystis *particles including dectin-1, β2 integrin CD11b/CD18, and lactosylceramide [16,17,23,24]. Airway epithelial cells specifically lack dectin-1 receptors, which are present in macrophages. Based on our recent observations demonstrating that lactosylceramide is responsible for MIP-2 production, we further evaluated the role of glycosphingolipids in cytokine signaling by airway epithelial cells activated with PCBG [17,18].

Herein, we demonstrate that 1HAEo^- ^human airway epithelial cells simulated with PCBG induce the release of the neutrophil chemokine IL-8, in a calcium-dependent manner. We further demonstrate the participation of two major MAPKs, ERK and p38, and that at least two major transcription factors, NF-κB and AP-1, are necessary for an adequate transcription of IL-8. Finally, we observed that glycosphingolipids are necessary for the synthesis of IL-8 by PCBG activated 1HAEo^- ^cells.

## Materials and methods

### Reagents and antibodies

Endotoxin-free buffers and reagents were scrupulously employed for all experiments. *Saccharomyces cerevisiae *derived cell wall β-glucans, the calcineurin disrupting agents TEMPO (2,2,6,6-Tetramethyl-1-piperidinyloxy, free radical, 2,2,6,6-Tetramethylpiperidine 1-oxyl) and cyclosporin B were purchased from Sigma Chemical Co, (St. Louis, MO). The calcium chelator BAPTA/AM (1,2-bis-(o-Aminophenoxy)-ethane-N,N,N',N'-tetraacetic acid, tetraacetoxymethyl ester) was obtained from Alexis Biochemical. The glucosylceramide synthase inhibitor PDMP (D-threo-1-Phenyl-2-decanoylamino-3-morpholino-1-propanol•HCl) was purchased from Matreya, LLC (Pleasant Gap, PA), LPS from *Escherichia coli *026:B6, EGTA, PD 98059, SB 202190, SB 202474, JNK inhibitor II and other general reagents were from Calbiochem (Gibbstown, NJ), unless otherwise specified. *Pneumocystis carinii *was derived originally from the American Type Culture Collection stock (Manassas, VA) and has been passaged though our immunosuppressed rat colony [25]. All antibodies employed in these studies were purchased from Cell Signaling Technologies (Danvers, MA). The human airway epithelial cell line, 1HAEo^- ^cells, were generously provided by Dr. Dieter Gruenert (University of California, San Francisco) [26]. The cells were routinely cultured in Modified Eagle's medium containing 10% fetal bovine serum and 2 mM L-glutamine, penicillin 10,000 units/liter, and streptomycin 1 mg/liter.

### Plasmids

The NF-κB-dependent firefly luciferase reporter expression vector (κB-luc) was a kind gift of Dr. Carlos Paya (Mayo Clinic, Rochester, MN)[27]. The IL-8, IL-8 mutated in AP-1, and NF-κB sites promoter-luciferase reporter plasmids were gifts from Dr. Marc Hershenson (University of Michigan)[28]. The pRL-TK expression vector, which provides constitutive expression of *Renilla *luciferase, was purchased from Promega (Madison, Wisconsin).

### Generation of *Pneumocystis carinii *β-Glucan-rich Cell Wall Isolate

The Mayo Institutional Animal Care and Usage Committee approved all animal experimentation. A β-glucan-rich cell wall fraction from *P. carinii *was prepared as we previously described [11,18]. *Pneumocystis *pneumonia was induced in dexamethasone-treated immunosuppressed Lewis rats (Harlan, Inc., Indianapolis, IN) [25]. *Pneumocystis *organisms were isolated from lungs of heavily infected animals by homogenization and filtration through 10-μm filters. The organisms were autoclaved (120°C, 20 min) and disrupted by ultrasonication (200 W for 3 min, six times), and the glucans were isolated by NaOH digestion and lipid extraction as previously detailed [11,18]. As we prior reported, the final product contained predominantly carbohydrate (95.7%) and released 82% of its content as D-glucose following hydrolysis [11]. Extensive measures were employed to ensure that the fractions were free of endotoxin. Prior to use in culture, the *Pneumocystis *cell wall fractions were washed with 0.1% SDS and then vigorously washed with distilled physiological saline to remove the detergent. The final preparation was assayed for endotoxin with the *Limulus *amebocyte lysate assay method and found to consistently contain < 0.125 units of endotoxin [11].

### IL-8 detection

IL-8 was measured in the supernatants of cultivated 1HAEo^- ^cells by ELISA (BD OptEIA™, BD biosciences, San Diego, CA). Cells were cultured to ~70% confluence in a 96-well plates. Prior to activation with PCBG, the cells were weaned from serum for 18 hours. For some experiments, the cells were preincubated with various calcium disrupting agents or MAPKs inhibitors for one hour prior to stimulation. Supernatant was collected after 8 hour of stimulation with PCBG unless otherwise indicated and stored at -70°C. All experiments were performed in duplicate and repeated on a minimum of at least three occasions.

### Cellular Viability

Cell viability was confirmed using the XTT Cell Proliferation Kit II (Roche Molecular Biochemicals, Mannheim, Germany). This assay measures the conversion of sodium-3'-[1-(phenylaminocarbonyl)-3,4-tetrazolium]-bis(4-methoxy-6-nitro) benzenesulfonic acid hydrate (XTT) to a formazan dye through electron coupling in metabolically active mitochondria using the coupling reagent *N*-methyldibenzopyrazine methyl sulfate. Only metabolically active cells are capable of mediating this reaction, which is detected by absorbance of the dye at 450-500 nm. Greater than 80% survival was considered acceptable cellular viability in all the experiments.

### Intracellular calcium flux determination using digital video fluorescence imaging

To measure intracellular Ca^2+ ^fluxes, cells were plated in 8 well borosilicate coverglass chambers and were incubated with 5 μM Fura-2AM (acetoxy-methyl-2-[5-[bis[(acetoxymethoxy-oxomethyl)methyl]amino]-4-[2-[2-[bis[(acetoxymethoxy-oxo methyl)methyl]amino]-5-methyl-phenoxy]-ethoxy]benzofuran-2-yl]oxazole-5-carboxylate, a calcium imaging dye that binds to free Ca^2+ ^in HBSS (Hanks balanced salt solution with 2.25 mM CaCl_2_, 0.8 mM MgSO_4 _and 12 mM glucose; pH 7.4) for 60 minutes at room temperature. Cells were then washed twice with fresh HBSS and subsequently maintained in HBSS. Cells were continuously perfused during the acquisition of Ca^2+ ^measurements. Fluorescence excitation, image acquisition, and Ca^2+ ^data analyses were controlled using a dedicated video fluorescence imaging system (Metafluor; Universal Imaging Corporation). Cells were imaged using an inverted Nikon Diaphot microscope equipped with a Nikon Fluor X20 objective lens. Fura 2-loaded cells were alternately excited at 340 and 380 nm using a Lambda 10-2 filter changer (Sutter Instrument Company). Fluorescence emissions were collected separately for each wavelength using a 510 nm barrier filter. Images were acquired using a Micromax 12 bit camera system (Princeton Instruments) approximately every 0.75 seconds. Intracellular Ca^2+ ^concentrations were calculated from the ratio of intensities at 340 nm and 380 nm, by extrapolation from a calibration curve as previously described [29]. For a positive control of intracellular calcium release, cells were stimulated in parallel with PAR-2 Peptide (Anaspec, San Jose, Ca (Protease activated receptor - 2)) at a final concentration of 100 μM.

### Cell extraction and immunoblotting

To obtain total cellular proteins, cells were washed with cold phosphate-buffered saline (PBS) twice and lysed in RIPA buffer (50 mM Tris-HCl pH 7.4, 15 mM NaCl, 0.25% deoxycholic acid, 1% NP-40, 1 mM EDTA) freshly supplemented with 2 μM phenylmethylsulfonyl fluoride [PMSF], 10 μg/ml aprotinin, 1 μg/ml leupeptin, 1 μg/ml pepstatin, 10 mM NaF and 300 μM Na orthovanadate. Cell lysates were centrifuged at 12,000 × *g *for 1 min at 4°C. The resultant supernatant contained total cellular protein. Protein concentrations in the clarified supernatants were determined using the Bio-Rad (Hercules, Calif.) protein assay. For Western immunoblotting, equal amounts of total cellular proteins were separated by 10% SDS-PAGE and transferred to Immobilon-P membranes (Millipore, Bedford, Mass.). Immunoblotting was performed with specific antibodies and visualized using the ECL enhanced chemiluminescence Western blotting detection kit (Amersham, Buckinghamshire, England). Densitometry analysis of the Immunoblots was performed using the computer program ImageJ 1.42d, National Institutes of Health, USA. The data was expressed as fold increase of the ratio between the protein of interest and the loading control.

### Gene transfection and reporter assays

Cells were seeded in 24-well plates. Lipofectamine Plus (Invitrogen) was used to transfect DNA plasmids into the 1HAEo^- ^cells according to the manufacturer's protocol. Following trasfection, the 1HAEo-cells were cultured for an additional 12 to 18 hours. Next, the cells were stimulated for eight hours with PCBG (100 μg/ml). One hour prior to stimulation, the cells were pretreated with PD98059 (16 μM), SB202190 (30 μM), JNK inhibitor II (10 μM) or BAPTA (1.2 μM). Following stimulation, the cells were washed twice in cold PBS and lysed with 50-100 μl of lysis buffer (Promega dual-luciferase reporter assay system). Firefly and *Renilla *luciferase activities from 10 μl of cell extracts were assayed with the Promega dual-luciferase reporter assay system reagents and a Berthold Lumat following the manufacturer's protocol. The κB-luc and IL-8 luc activities were normalized for *Renilla *expression. All transfection experiments were performed in duplicate.

### Effects of glycosphingolipids inhibitors on PCBG induced IL-8 secretion and ERK phosphorylation by airway epithelial cells

Cells were cultured as previously described, and incubated with PDMP to reduce glycosphingolipid concentration, or media alone, for 72 hours prior to PCBG stimulation. Phosphorylation of p44/42 was analyzed from total cell lysates by immunoblotting and IL-8 was measured by ELISA in the culture supernatant. To exclude toxicity to the airway epithelial cells induced by PDMP, XTT viability assays were performed under identical conditions. Greater than 80% viability was considered as acceptable cellular viability for all experimental conditions.

### Statistical and data analyses

All data are shown as the means ± SEM, unless otherwise stated. Data were assessed for significance using the Student *t *test or ANOVA with relevant posttests where appropriate. Statistical differences were considered to be significant if *p *was < 0.05. Statistical analysis was performed using GraphPad Prism version 5 (GraphPad Software, La Jolla, CA).

## Results

### PCBG induce IL-8 secretion from 1HAEo-cells

**Figure 1:**
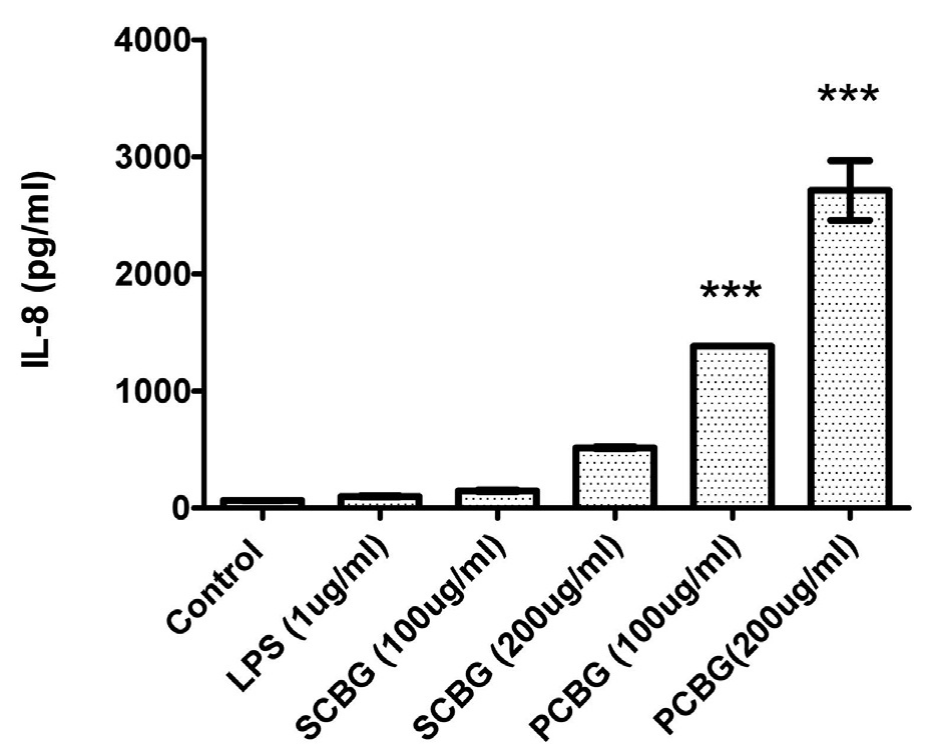
**PCBG induces IL-8 release from 1HAEo^- ^human airway epithelial cells**. Cells were incubated with LPS, *Saccharomyces cerevisiae *β-glucan and *Pneumocystis *β-glucan at the indicated doses for a period of 14 hours. Release of IL-8 was measured by ELISA in the media supernatant of the cells. Data were analyzed with one-way ANOVA and posttest Dunnett's comparison test (*** denotes *p *< 0.001). The experiment shown is representative of three independent experiments.

Since patients with severe *Pneumocystis *pneumonia exhibit an intense neutrophil infiltration in their lungs, we postulated that airway epithelial cells might participate in IL-8 secretion and subsequent recruitment of inflammatory cells in response to infection [5,30,31]. Our prior studies have been performed in rat primary alveolar epithelial cells [17]. However, such primary cell cultures are of rodent origin and, as primary cultures, have limited ability to evaluate signaling pathways and promoter mechanisms. Therefore, in this investigation we utilized the 1HAEo-human airway epithelial cell line. Accordingly, we first determined whether IL-8 was secreted by 1HAEo-airway epithelial cells challenged with either PCBG or *S. cerevisiae *derived β-glucans. The 1HAEo^- ^cells were exposed to the fungal β-glucan preparations, or LPS, and IL-8 release was measured after 14 hours of challenge. *P. carinii *and to a lesser degree *Saccharomyces *derived β-glucans induced IL-8 secretion in a dose-dependent manner compared with both unstimulated and LPS challenged cells (Figure 1). Significantly, the absence of response of these cells to LPS excluded the possibility that endotoxin contamination of the β-glucan preparation was responsible for the observed inflammatory responses.

### IL-8 secretion by airway epithelial cells stimulated with PCBG is calcium-dependent

**Figure 2:**
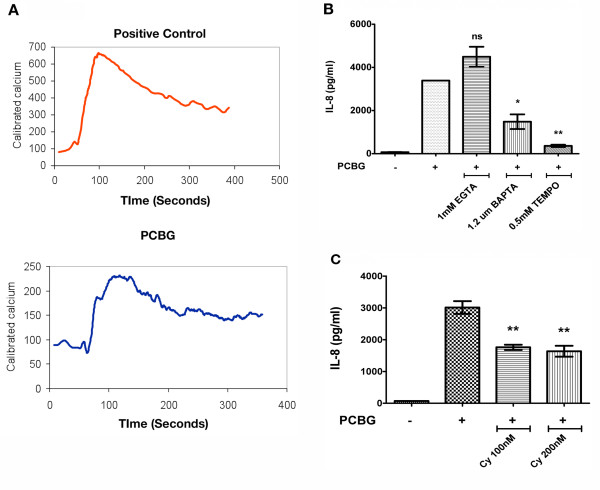
**Intracellular calcium mobilization after PCBG stimulation**. **A**. Airway epithelial cells (1HAEo-cells) were loaded with Fura-2AM and incubated with either 100 ug/ml of PCBG or with PAR-2 Peptide control (100 μM) for the indicated times and transient intracellular calcium release monitored by video fluorescence imaging. **B**. In additional experiments, airway epithelial cells were incubated with 100 ug/ml of PCBG. For one hour prior to the addition of PCBG, the cells were preincubated with various calcium and calcineurin disrupting agents (EGTA, BAPTA, or TEMPO) at the concentration indicated. IL-8 secretion was measured by ELISA in the supernatant of the cells after eight hours of incubation. **C**. Finally, airway epithelial cells were incubated with 100 ug/ml of PCBG for eight hours in the presence of cyclosporine A at the indicated concentration and IL-8 secretion measured by ELISA. Data were analyzed with one-way ANOVA and posttest Bonferroni comparison (*denotes *p *< 0.05; **denotes *p *< 0.01). The data shown are representative of three independent experiments.

Since various microbial ligands are able to initiate intracellular calcium fluxes during cell stimulation, we next investigated whether PCBG challenge of airway epithelial cells triggered intracellular calcium release [31,32]. Consistent with this, we observed that PCBG-treated cells release intracellular calcium within a few seconds of stimulation (Figure 2A). As a positive control, a potent PAR-2 agonist peptide was tested in parallel. The peak wave of calcium release in PCBG treated cells appeared to be somewhat slower and maybe more prolonged than in PAR-2 treated cells. We believe that this is explained by the differences in formulation between the two compounds. While PAR-2 is a soluble reagent, and likely acts quicker on the cells, PCBG is a particulate agonist with slower action time.

Next, we sought to evaluate the importance of calcium release in IL-8 secretion of PCBG stimulation of 1HAEo^- ^epithelial cells. Accordingly, cells were pretreated with various calcium-signaling disrupting agents prior to PCBG stimulation and IL-8 release was determined in the culture supernatants, after 8 hours of stimulation (Figure 2B and 2C). Cells pretreated with EGTA, an extracellular calcium chelator [33], did not demonstrate any decrease in IL-8 secretion. In contrast, epithelial cells preincubated with the intracellular chelator BAPTA/AM [34], the calcineurin disrupting agents TEMPO, or cyclosporin A [35] each demonstrated significant decrease in IL-8 production (Figure 2B and 2C). Together, these data indicate that optimal secretion of IL-8 by airway epithelial cells stimulated with PCBG requires intra-cellular, rather than extra-cellular, calcium mobilization.

### IL-8 secretion by airway epithelial cells is mediated by NF-κB and AP-1

**Figure 3:**
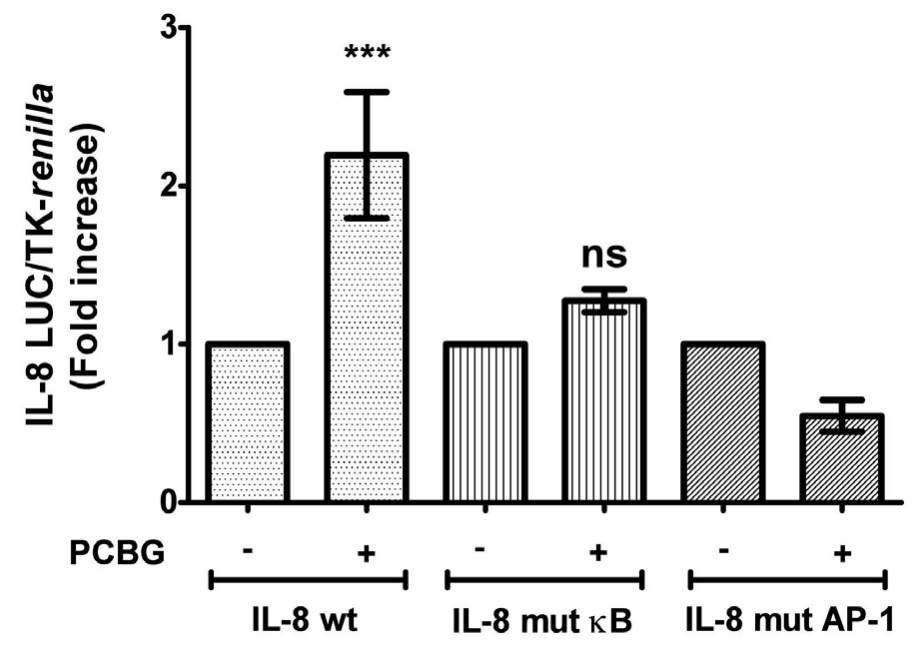
**PCBG induced IL-8 expression requires NF-κB and AP-1 activation**. 1HAEo^- ^cells were transiently transfected with the IL-8 promoter (WT), the IL-8 promoter mutated at the NF-κB site (mut kB) or the IL-8 promoter mutated at the AP-1 site (mut AP-1). TK-*renilla *(10 ng) was co-transfected as an internal control as indicated in material and methods. Eighteen hours later, transfected 1HAEo^- ^cells were challenged with 100 ug/ml of PCBG. After an additional eight hours of incubation, the cells were harvested and luciferase activities were measured. The IL-8 activity was normalized to *Renilla *luciferase activity (relative lights units). Data were analyzed with one-way ANOVA and posttest Bonferroni comparison (***denotes *p *< 0.01). The data shown is the average of two independent experiments.

A variety of transcription factors including NF-κB and AP-1 binding sites have been identified within the IL-8 promoter [36-42]. These transcription factors bind the promoter as dimers, and various combinations of AP-1 and NF-κB have been shown to be important for optimal activation of the IL-8 promoter, particularly in epithelial cells [43]. Therefore, to further investigate the importance of NF-κB and AP-1, in IL-8 production induced by β-glucans, we measured IL-8 activation in 1HAEo-cells transiently transfected with the IL-8 luciferase reporter construct or with an IL-8 luciferase reporter construct that had targeted mutations in the NF-κB or AP-1 binding sites (Figure 3). PCBG failed to activate IL-8 transcription in cells transfected with either the mutant NF-κB or mutant AP-1 constructs, whereas IL-8 transcription was activated normally in cells transfected with the wild-type IL-8 promoter construct. From these observations, we can imply that both transcription factors are necessary for optimal activation of IL-8 transcription by airway epithelial cells following stimulation with PCBG.

### IL-8 secretion by PCBG stimulated airway epithelial cells is mediated by MAP Kinases

**Figure 4:**
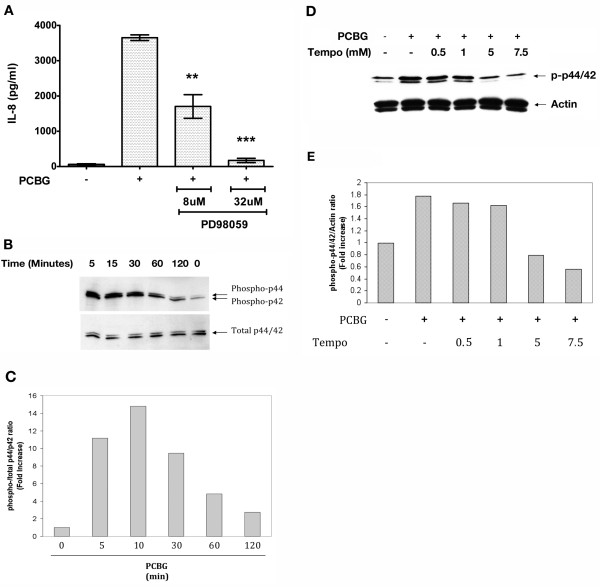
**PCBG induces activation of ERK in 1HAEo^- ^airway epithelial cells**. **A**. 1HAEo^- ^cells were challenged with 100 ug/ml of PCBG for eight hours and IL-8 release assessed by ELISA in the culture supernatants. Cells were pretreated for 1 hour with the ERK inhibitor PD 98059 or vehicle solution as indicated prior to the addition of PCBG. Data were analyzed with one-way ANOVA and posttest Bonferroni comparison (**denotes *p *< 0.01; ***denotes p < 0.001). **B**. 1HAEo^- ^cells were incubated with 100 ug/ml of PCBG for the indicated times, and phospho-p44/p42 and total p44/p42 were detected by western blot in the total cell lysate. **C**. Densitometry analysis of phospho- p44/p42 to total-p44/p42 ratio. **D**. 1HAEo^- ^cells were pre-incubated for 1 hour with different concentrations of TEMPO prior to stimulation with 100 ug/ml of PCBG for 10 minutes, phospho-ERK p44/p42 was detected by Western blot in the total cell lysate. Actin was shown as loading control. **E**. Densitometry analysis of phospho- p44/p42/Actin ratio. The data shown is representative of at least two independent experiments.

Since MAPKs has been implicated in IL-8 secretion by airway epithelial cells, we next investigated whether MAPK activation was necessary for β-glucan stimulation of airway epithelial cells to release IL-8 [31,44,45]. To accomplish this, 1HAEo^- ^cells were preincubated with PD98059, a specific pharmacological inhibitor of ERK, prior to stimulation with PCBG. Cells pre-treated with PD98059 exhibited a dose-dependent decrease in IL-8 production in response to the PCBG compared with untreated cells (Figure 4A). To further understand the kinetics of MAPK/ERK activation phosphorylation of ERK was determined by western immunoblotting after stimulation of the cells for different periods of time as indicated in Figure 4B. Phosphorylation of ERK p44/42 was detected within five minutes of stimulation, and remained slightly elevated as long as two hours after the initial challenge (Figure 4B and 4C). In addition, the calcineurin-disrupting agent TEMPO impaired ERK phosphorylation (Figure 4D and 4E).

**Figure 5:**
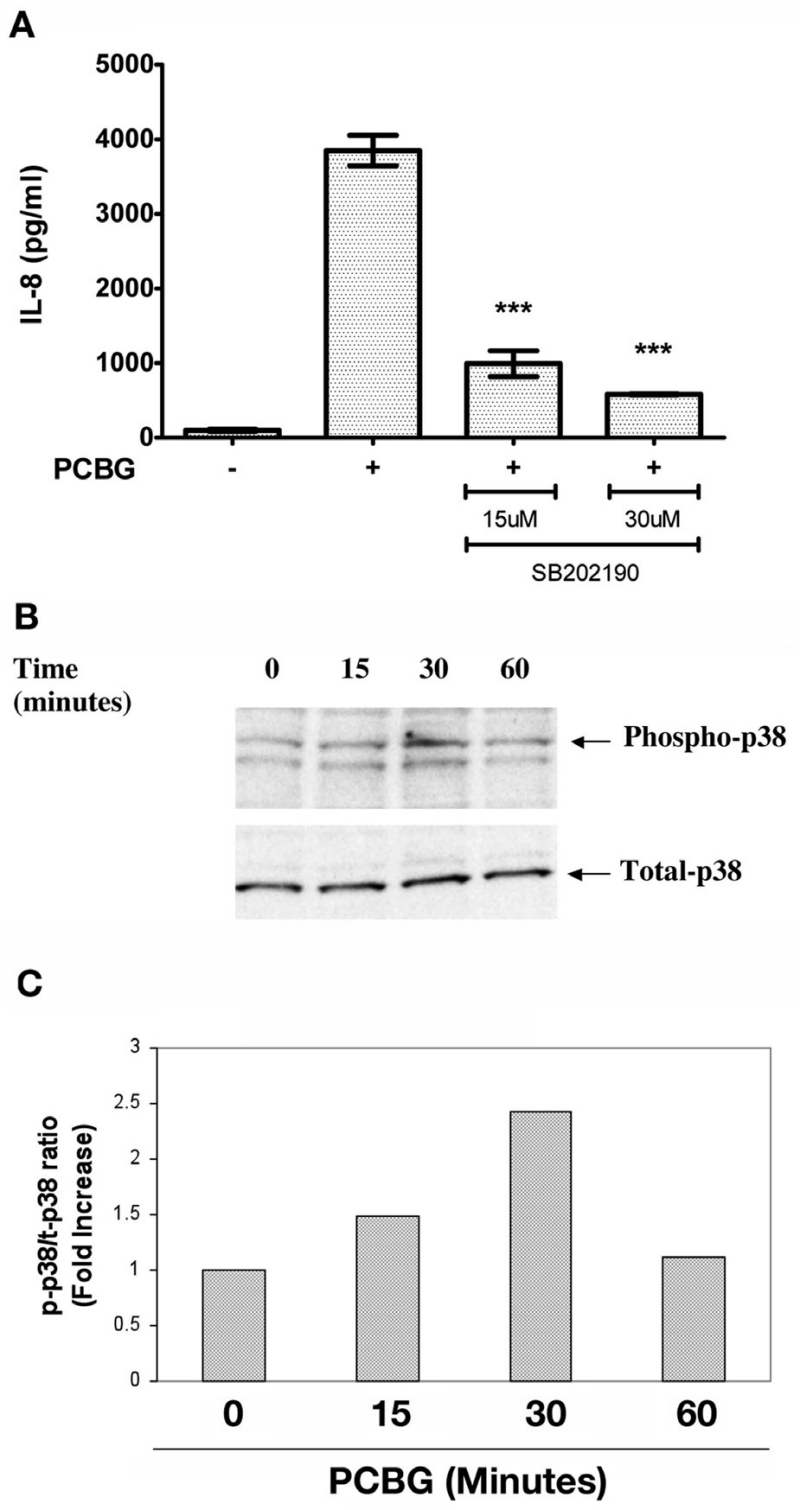
**Activation of p38 MAPK after PCBG stimulation of 1HAEo-cells**. **A**. 1HAEo^- ^cells were incubated with 100 μg/ml of PCBG for a period of eight hours, and the media supernatants collected and IL-8 measured by ELISA. Prior to the addition of PCBG the cells were pretreated for 1 hour with the p38 inhibitor SB202190. Data were analyzed with one-way ANOVA and posttest Bonferroni comparison (***denotes p < 0.001). **B**. 1HAEo^- ^cells were challenged with 100 μg/ml of PCBG for the times indicated and phospho-p38 and total p38 analyzed by western blot in the total cell lysates. C. Densitometry analysis of phospho-p38 to total p38 ratio. The data shown is representative of three independent experiments.

Next, we evaluated whether p38, an independent major MAPKs pathway, participated in β-glucan mediated IL-8 secretion from airway epithelial cells in response to PCBG (Figure 5). The specific pharmacological inhibitor of p38, SB202190, was administered prior to and throughout PCBG stimulations of 1HAEo^- ^cells. Notably, SB202190 treated cells demonstrated significant reduction of IL-8 secretion in a dose-dependent manner, indicating the participation of p38 in the release of IL-8 (Figure 5A). In addition, we further investigated the kinetics of p38 activation following PCBG stimulation. Phosphorylation of p38 was detected as early as 15 minutes following stimulation, and reached its peak after 30 minutes. Following one hour of PCBG stimulation, phosphorylation of p38 had returned to baseline levels (Figure 5B and 5C). These data verify differential kinetics of these two MAPK signaling pathways, with the activation of p38 being substantially slower than the phosphorylation of ERK p44/42.

**Figure 6:**
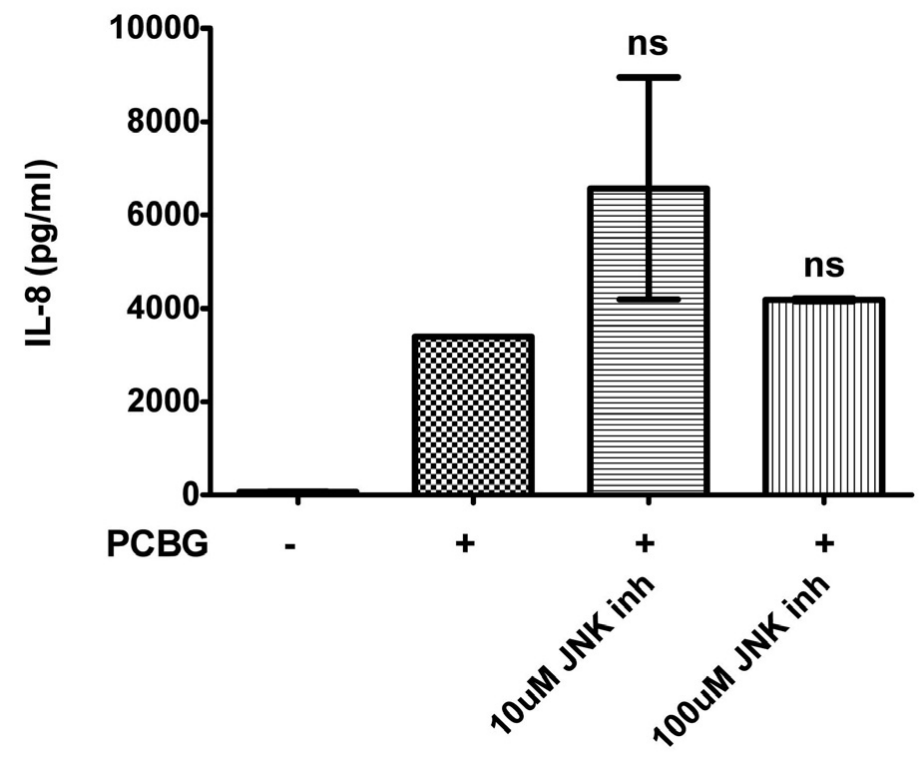
**IL-8 production by PCBG activated cells is not impaired in the presence of a pharmacological inhibitor of JNK-II**. 1HAEo^- ^cells were incubated with 100 ug/ml of PCBG for a period of eight hours. Prior to the addition of PCBG, the cells were preincubated for one hour with JNK Inhibitor II at the concentration indicated. IL-8 secretion was measured by ELISA in the media supernatant of the cells. Data were analyzed with one-way ANOVA and posttest Bonferroni comparison (not significantly different, *p *> 0.05). The data shown is representative of two independent experiments.

Finally, we investigated whether another important member of the MAPK signaling family, JNK, was also involved in IL-8 secretion by airway epithelial cells following challenge with PCBG (Figure 6). The JNK inhibitor II, a pharmacological antagonist of JNK was used prior to and through stimulation of 1HAEo-cells over PCBC for eight hours [46]. Interestingly, we did not detect any inhibition of IL-8 secretion in PCBG stimulated cells in the presence of the JNK-II inhibitor. To verify that the inhibitor was functionally active, we further analyzed phosphorylation of JNK in PCBG stimulated cells in the presence of JNK inhibitor II in comparison to cells that were stimulated with PCBG in the absence of the inhibitor, verifying that JNK phosphorylation was indeed greatly reduced (data not shown). Nevertheless, IL-8 secretion was not impacted by this inhibitor, indicating that the participation of ERK and p38 MAPK in airway epithelial cells stimulated with PCBG is specifically restricted to those pathways, and that JNK does not participate in this cytokine response.

### MAPK activation in PCBG stimulated 1HAEo^- ^cells stimulates downstream NF-κB expression

**Figure 7:**
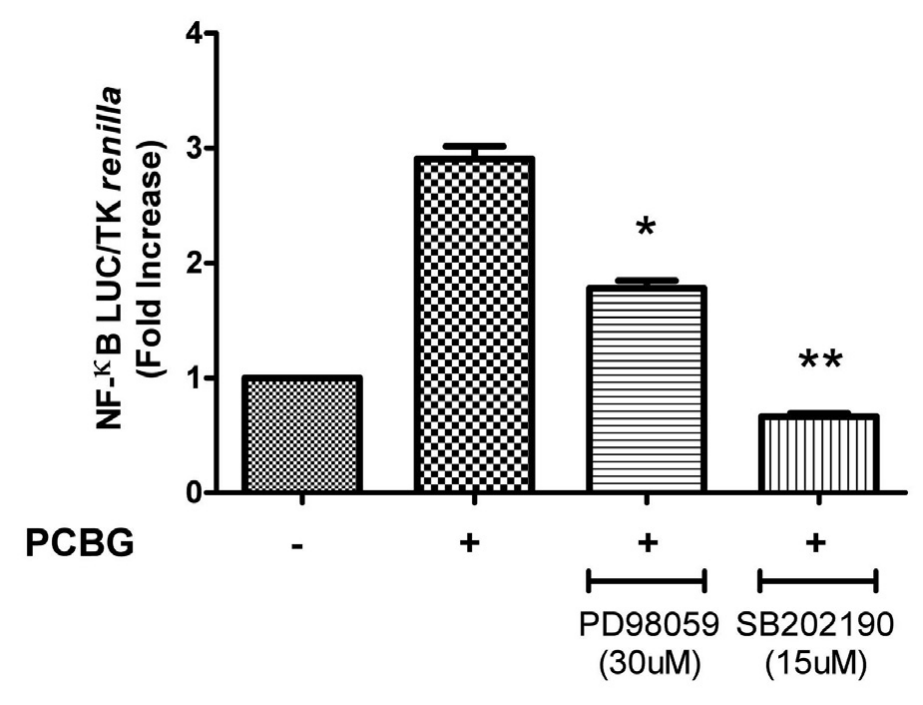
**NF-κB activation is impaired in the presence of MAPKs inhibitors and an intra-calcium chelator, but not in the presence of JNK inhibitor**. 1HAEo^- ^cells were transiently transfected with the NF-κB reporter (50 ng) and TK-*renilla *(10 ng) as indicated in the Material and Methods. Eighteen hours later, transfected 1HAEo^- ^cells were challenged with 100 μg/ml of PCBG, prior stimulation the cells were preincubated for 1 h with the different inhibitors. Eight hours later, the cells were harvested and luciferase activities were measured. The NF-κB activity was normalized to *Renilla *luciferase activity (relative lights units). Data were analyzed with one-way ANOVA and posttest Bonferroni comparison (*denotes *p *< 0.05; **denotes *p *< 0.01). The data shown is representative of three independent experiments.

We have previously shown that MIP-2 neutrophil chemokine induced by PCBG in rodent primary lung epithelial cells is mediated by NF-κB activation (10). We next sought to determine whether MAPK activation following β-glucan stimulation of human 1HAEo^- ^cells resulted in downstream NF-κB dependant activation (Figure 7). To test this, we evaluated whether PCBG induced ERK and p38 signaling resulted in NF-κB promoter dependent activation in 1HAEo^- ^cells that were transiently transfected with an NF-κB-dependent luciferase reporter plasmid. Prior to PCBG stimulation, the 1HAEo^- ^cells were incubated with either; the PD98059, SB202190, or the JNK inhibitor II. Notably, pre-incubation of the cells with either PD98059 or SB202190 significantly reduced NF-κB dependent transcriptional activity in PCBG stimulated cells. However, the addition of JNK inhibitor II again had no effect on transcriptional activity related to NF-κB. These data suggest that PCBG mediated MAPKs activation results in downstream NF-κB-dependent transcriptional activation in target airway epithelial cells.

### Inhibition of glycosphingolipids synthesis further impairs IL-8 released by airway epithelial cells stimulated with PCBG

**Figure 8:**
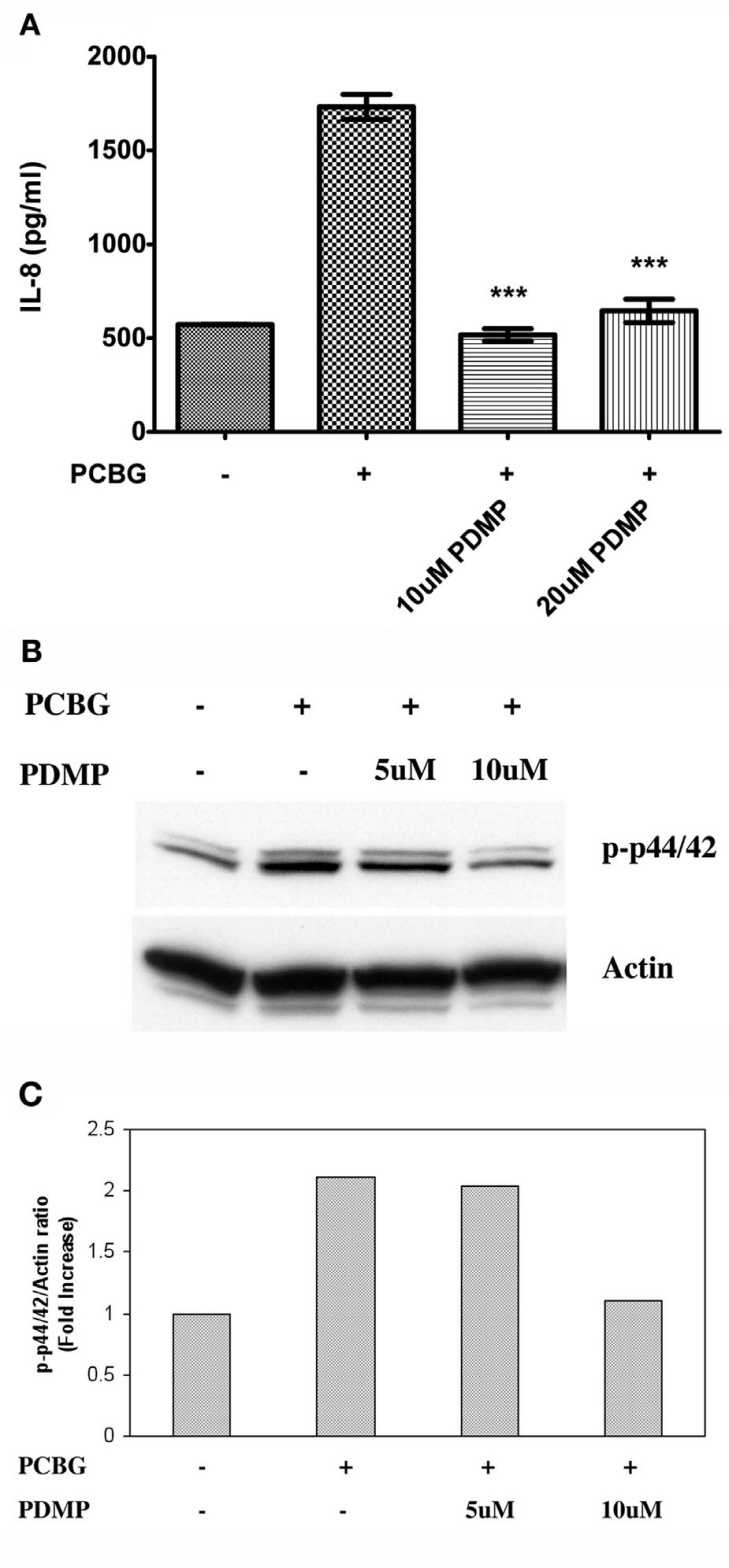
**Effect of glycosphingolipid synthesis inhibitors on PCBG-mediated IL-8 secretion from 1HAEo-airway epithelial cells**. **A**. Cells were incubated with different concentrations of PDMP for 72 hours prior to stimulation with 100 ug/ml of PCBG, and the cells incubated an additional 14 hours. Supernatants were assayed for IL-8 as described. Data were analyzed with one-way ANOVA and posttest Bonferroni comparison (***denotes p < 0.001). The data shown is representative of three independent experiments. **B**. **1HAEo**^**- **^cells were incubated for 72 hours in the presence of PDMP at the concentrations indicated, or media alone prior to stimulation with PCBG for 30 min. Phospho-p44-42 was analyzed by western blot and actin was assessed in parallel to verify equal loading. C. Densitometry analysis of phospho- p44/p42 to Actin ratio.

Previous data from our laboratory indicate that PCBG requires the glycosphingolipid lactosylceramide to induce MIP-2 release in murine epithelial cells [17,47]. We, therefore, sought to determine whether IL-8 secretion by PCBG in these human airway cells was also dependent on the presence of glycosphingolipids. To accomplish this, we evaluated IL-8 secretion in PCBG stimulated cells in the presence of PDMP, a potent glycosphingolipid synthesis inhibitor. Serum free media cultivated cells were treated with PDMP for 3 days prior to stimulation with PCBG. IL-8 release from β-glucan stimulated airway epithelial cells treated with the glycosphingolipid inhibitor was significantly decreased compared to non-treated cells (Figure 8A). We further investigated the effect of PDMP on ERK phosphorylation. Cells were cultured with media alone or in the presence of PDMP prior to activation with PCBG. Total cell lysates were analyzed for phospho-p44/42 by immunoblotting (Figure 8B and 8C). The phosphorylation of ERK p44/42 was reduced to baseline in cells treated with PDMP compared with non-treated cells. Taken together, these data strongly support our findings that glycosphingolipids are important for PCBG mediated ERK activation and subsequent IL-8 secretion by airway epithelial cells in response to PCBG.

## Discussion

Tissue inflammation is an essential component of host defense against infection, however, exaggerated inflammatory response can be extremely deleterious to the host. Considerable evidence reveals this to be particularly true for *Pneumocystis *pneumonia. Early studies from our laboratory, as well as from other investigators have documented that death and respiratory failure in patients with *Pneumocystis *pneumonia is largely related to the intense inflammatory reaction induced by the infection rather than direct toxic effects of the fungus [3-5,9,30]. Many patients with this infection present with intense neutrophilic and CD8 lymphocytic infiltration in the lungs and associated impaired oxygen exchange. What induces the exaggerated recruitment of inflammatory cells in these patients remains poorly understood. These studies were undertaken to address the molecular mechanisms, which regulates the potent neutrophil chemoattractant factor, IL-8 in airway epithelial cells challenged with the potent pro-inflammatory cell wall component of *Pneumocystis *β-glucan.

Studies from our lab have documented the inflammatory properties of PCBG, and have revealed that this carbohydrate-rich cell wall fraction is capable of inducing specific chemokines and cytokines in cells such as macrophages, dendritic cells (DC) and alveolar epithelial cells [11,12,17,18]. Airway epithelial cells are the first cells to come into contact with inhaled pulmonary pathogens. Contrary to earlier beliefs that alveolar epithelial cells were only involved in gas exchange, emerging evidence has documented the importance of these cells as a rich source of inflammatory mediators, particularly chemokines. We have specifically demonstrated that rodent alveolar epithelial cells undergo NF-κB mediated MIP-2 release when challenged with *Pneumocystis *β-glucans. In this regard, airway epithelial cells exhibit greater potency than alveolar macrophages challenged with this cell wall component (10, 19). In the present study, we further demonstrate that human airway epithelial cells secrete significant amounts of IL-8, the human homologue of MIP-2, in response to *Pneumocystis *cell wall β-glucan. We have further observed that airway epithelial cells mobilize intracellular calcium within seconds following β-glucan stimulation. This intra-calcium flux initiates the activation of the two major MAPKs pathways, ERK and p38, and subsequent activation of AP-1 and NF-κB, resulting in the release of IL-8. Finally, we demonstrated that inhibition of glycosphingolipids synthesis significantly impairs the IL-8 response of these cells, suggesting an important role for surface membrane glycosphingolipids conferring inflammatory activation.

Glycosphingolipids, most notably lactosylceramide, have been proposed as receptors for fungal β-glucans, and have been of particular interest in cellular activation mediated by *Pneumocystis *(15, 16). In the present study, we demonstrated that treatment of human airway epithelial cells with PDMP, a glycosphingolipid synthesis inhibitor, dramatically reduced the ability of *Pneumocystis *β-glucans to stimulate IL-8 release, strongly indicating that glycosphingolipids are important components initiating epithelial cell signaling. In the present study, we further observed that intracellular calcium mobilization, as well as activation of two major MAPK pathways (ERK and p38), also participate in epithelial cells responses to PCBG.

Intracellular calcium mobilization appears necessary for IL-8 secretion, since PCBG does not activate airway epithelial cells in the presence of the intracellular calcium chelator BAPTA/AM or the calcineurin inhibitor TEMPO. This early intracellular mobilization of calcium acts through additional second messengers to induce activation of the ERK and p38 MAPK pathways. Interestingly, these two pathways are likely stimulated through unique mechanisms, since their kinetics of activation were significantly different. While ERK p42/44 was phosphorylated within five minutes of stimulation, p38 reach its peak phosphorylation after 30 minutes. Ultimately, ERK and p38 pathways were both found to impact downstream NF-κB activation at the transcriptional level.

In contrast, we did not observe any decrease in IL-8 levels nor NF-κB transcriptional activation in the presence of the specific pharmacological inhibitor of JNK, suggesting that JNK does not participate in PCBG induced cell stimulation. Recently, an interesting report by Wang and coworkers demonstrated that whole *Pneumocystis *induced the release of MCP-1 from alveolar epithelial cells in a JNK-dependent fashion that did not appear to require β-glucan [48]. The study of Wang and colleagues utilized β-glucan derived from *S. cerevisiae *[48]. While we observed some minimal activation of epithelial cells by *Saccharomyces *β-glucan, PCBG was shown to be far more potent in stimulating the epithelial cells in a JNK independent manner in our hands.

The observations of our current study are comparable to those of Slevogt and coworkers, who noted activation of ERK and p38 but not participation of JNK in *Moroxella catarrhalis *induced IL-8 production by epithelial cells [49]. Interestingly, other studies have revealed differing patterns of MAP activation in response to other microorganisms. For instance, Lamont and coworkers has shown that *Porphyromonas gingivalis *infection of epithelial cells is associated with JNK activation, down regulation of ERK and NF-κB activation, and decrease of IL-8 expression [50]. These studied support the notion that species-specific stimuli result in specific, and often differing, cellular IL-8 responses. In the case of *Pneumocystis*, two predominant pathways appear to augment IL-8 responses and neutrophilic recruitment in this pneumonia.

Regulation of IL-8 transcription is mediated by various transcription factors including NF-κB, AP-1, and NF-IL-6, which appear to be both stimuli and cell type specific [51]. For instance, adequate induction of IL-8 by TNF-α stimulated epithelial cells requires AP-1 and NF-κB binding activity to the IL-8 promoter, while AP-1 binding activity does not appear to be necessary in TNF-α stimulated endothelial cells [43]. This same group of investigators also demonstrated that AP-1 binding, and not the NF-κB, is critical for IL-8 expression by H_2_O_2 _stimulated epithelial cells [43]. The current studies demonstrate that IL-8 secretion and gene transcription induced by PCBG in human airway epithelial cells requires the integrity of NF-κB and AP-1 binding sites. This is noteworthy, because distinct AP-1 dimers may selectively interact with various NF-κB subunits and synergistically act to augment IL-8 expression. Such interaction have been demonstrated to occur in respiratory syncytial virus (RSV) induced IL-8 expression [41]. Indeed, in RSV infected cells, AP-1 cooperates preferentially with NF-κB, while in TNF-α stimulated cells NF-IL-6 interacts with NF-κB [41]. Based on these observations, we postulate that alteration of the binding between these various transcription factor subunits may help to initially promote IL-8 secretion in *Pneumocystis *pneumonia and subsequently to control neutrophil inflammation in this infection.

## Conclusion

In summary, our investigations have demonstrated that *Pneumocystis *cell wall β-glucans induce inflammatory response in human airway epithelial cells. IL-8 secretion by these cells involves membrane glycosphingolipid receptors and the intracellular mobilization of calcium, with subsequent phosphorylation of MAPKs pathways including ERK and p38. These events lead to downstream activation of the NF-κB and AP-1 transcription factors and ultimately to IL-8 release. Abrogation of either one or these MAPK pathways or these transcription factors results in a blunted IL-8 response. Better knowledge of the molecular mechanisms regulating chemokine generation will be essential to understand the recruitment of inflammatory cells to the lung during *Pneumocystis *pneumonia, and to design new treatment strategies for the exuberant lung inflammation that accompanies this infection.

## Competing interests

The authors declare that they have no competing interests.

## Authors' contributions

EMC performed the cytokine, signal transduction, and promoter assays and participated in drafting the manuscript. JDL assisted with the signal transduction assays and cell culture work. AX participated in the calcium signaling studies. MW participated in its design and coordination of the calcium experiments. AHL participated in the overall experimental design concept, review and interpretation of data, preparation of the manuscript and secured all funding for these studies. All authors read and approved the final manuscript.
